# A new tooth brushing approach supported by an innovative hybrid toothbrush-compared reduction of dental plaque after a single use versus an oscillating-rotating powered toothbrush

**DOI:** 10.1186/s12903-018-0647-7

**Published:** 2018-11-06

**Authors:** D. Klonowicz, M. Czerwinska, A. Sirvent, J-Ph. Gatignol

**Affiliations:** 1 Dermscan Poland, Ul. Kruczkowskiego 12, 80-288 Gdansk, Poland; 2 Laboratoire Dermscan, 114 Bd du 11 novembre 1918, 69100 Villeurbanne, France; 3 Innovation Unit Consumer HealthCare, 17 avenue Jean Moulin, 81106 Castres Cedex, France

**Keywords:** Powered toothbrush, Sonic toothbrush, Oscillating-rotating toothbrush, Dental plaque, Manual toothbrush, Hybrid toothbrush

## Abstract

**Background:**

An innovative hybrid toothbrush was designed functioning either in manual mode, in powered mode (sonic) or in combined mode (manual and powered). The primary aim of this study was to evaluate and compare the clinical efficacy of this first hybrid toothbrush (*Elgydium Clinic/Inava Hybrid*) used in combined mode to a marketed oscillating-rotating powered toothbrush (*Oral-B Vitality*) in the reduction of dental plaque after a single use. The secondary aims were to evaluate the tolerance and acceptability of each device.

**Methods:**

It was a randomized, examiner-blind, single-center study performed on two parallel groups: hybrid toothbrush (*n* = 33) versus oscillating-rotating toothbrush (n = 33). A brushing exercise was conducted for two minutes on subjects presenting a “Silness and Löe Plaque Index” (PI) between 1.0 and 2.0 and a “Modified Gingival Index” between 1.0 and 2.0. They were not to have ever used an electric toothbrush. To assess the device effect after brushing, a paired t-test was applied on the change outcome (After-Before brushing). An unpaired t-test was used to compare the efficacy of both devices. A global tolerance assessment of each powered toothbrush was done on all the subjects. The number and percentage of reactions related to each toothbrush was collected and the final tolerance assessment was estimated.

**Results:**

After a single use, the hybrid toothbrush used in combined mode presented a global anti-plaque efficacy characterized by a significant decrease of the global PI of 45% on average (*p* < 0.0001; paired t-test). It was as effective as the oscillating rotating toothbrush in plaque removal (*p* > 0.05; unpaired t-test). The global tolerance of both toothbrushes was judged as “Good” and they were equally appreciated by the users.

**Conclusion:**

The results of this one-time use trial demonstrate the efficacy of the hybrid toothbrush used in combined mode for plaque removal. The hybrid toothbrush design allows each user to adapt tooth brushing to his preference (manual / sonic / combined), his skills or his mouth condition. We hypothesize that such an individualized approach can favor long term compliance with oral health recommendations and improve global oral wellness.

**Trial Registration:**

ISRCTN12394494, 20/02/2018 - Retrospectively registered.

## Background

Oral health is one of the major concerns of dental health care professionals [1]. Most of periodontal diseases (halitosis, gingivitis, periodontitis, gum abscesses, peri-implant mucositis, etc.) are bacteria-dependent, through biofilm formation and dental plaque accumulation [2]. Promotion of regular oral hygiene can contribute to the maintenance of a functional dentition throughout life [3]. Current available approaches to control bacterial plaque development can be categorized as either mechanical (toothbrushes, floss, interdental brushes, chewing sugar-free gums) or chemotherapeutical (toothpastes, mouthwashes, gels) [4]. In Western industrialized countries, the toothbrush is widely accepted as a simple, affordable and effective device to remove plaque in a shorter time [5, 6]. To date, dental professionals recommend brushing teeth twice a day for two minutes [7, 8]. However, a wide diversity in brushing methods does exist depending on the position and motion of the brush. There appears to be no consensus on the ideal technique neither for the general population nor for people of different ages or with particular dental conditions [9]. No method is satisfactory when considering interdental cleanliness. Tooth brushing is able to clean the buccal, lingual and occlusal tooth surfaces but the proximal and interdental areas are often stayed untouched [10] or roughly cleaned [11]. Furthermore, tooth brushing efficacy for plaque removal relies on several parameters: motivation and skills of the subject, the use of a brush that fits the mouth allowing it to reach all areas, as well as proper oral hygiene education with instructions on movement, duration and frequency of brushing [12]. As an example, the two-minute recommendation for brushing time is hardly ever reached in behavioral studies. Most people spend between 30 and 60 s brushing their teeth [13, 14], while plaque removal efficacy is known to be time-dependent [15–17].

Powered toothbrush was conceived in the 1950s with a view to improve and facilitate oral hygiene. It was designed to target patients with limited motor skills as well as orthodontic patients, who have difficulties in keeping their teeth hygienic and healthy [18]. Geriatric patients with impaired manual skills can also benefit from powered toothbrushes [19]. Since the 1980s, powered toothbrushes have rapidly developed to become an established alternative to manual tooth brushing [20, 21]. Tremendous work has been done in order to improved toothbrush design, head and bristles. Several modes of action can be found on the market (oscillation-rotation, side-to-side sonic action, counter oscillation, circular, ultrasonic, ionic) but two technologies are dominating: oscillation-rotation and sonic. With the former, a small round brush head rotates in one direction and then the other, with the latter, a traditional brush head moves laterally form side to side with a high vibrational speed (mean frequency range 250 Hz, i.e. 30,000 brush-strokes-per-minute). This latter agitates the fluids present in the mouth (water, saliva) to the degree that they are able to disrupt dental plaque colonies even beyond where the bristles of the brush actually touch [22–24]. This fluid-dynamics cleaning action, although considered as a secondary cleaning effect, is characteristic of sonic toothbrushes.

A new generation of toothbrush called hybrid toothbrush has been recently developed. Hybrid toothbrush means that it can be used either in manual mode (motor off), in powered mode (sonic) or in combined mode (manual gesture associated to sonic vibrations). These various modes allow the user to adapt tooth brushing to his desire or his mouth condition. However, the use of combined mode corresponds to a new way of brushing. Indeed, usual recommendations for sonic tooth brushing imply minimal hand movements. In this case, hybrid toothbrush could be used by applying slight pressure by slowly moving the brush head with a light circular motion.

The primary aim of this study was to evaluate and compare the clinical efficacy of the first hybrid toothbrush (*Elgydium Clinic/Inava Hybrid*) using combined mode to a marketed oscillating-rotating powered toothbrush (*Oral-B* *Vitality*) in the reduction of dental plaque after a single use. The secondary aims of the study were to evaluate the tolerance and acceptability of each device.

## Methods

### Experiment

The experiment was a randomized, examiner-blind, single-center study performed on parallel group. It took place at Dermscan Poland (Gdansk, Poland). In Poland, an electric toothbrush is considered to be a domestic electric apparatus. Conducting a clinical study with such devices does not require any approval by an ethics committee. However, the study was conducted in compliance with Good Clinical Practices and in accordance with the “Declaration of Helsinki.” Written informed consents for participation in the clinical study were obtained for all participants.

### Subjects

The planned number of subjects to be analyzed was 30 minimum per group. In order to participate in the study, subjects had to be aged between 18 to 70 years old, to present at least 20 natural teeth, without implants, prosthesis or dental braces on the studied teeth. To qualify for the study, subjects had to present a “Silness and Löe Plaque Index” between 1.0 and 2.0 and a “Modified Gingival Index” between 1.0 and 2.0. They were not to have ever used an electric toothbrush. Subjects having undergone surgery, chemical or physical treatment in the mouth in the last 3 months as well as subjects using preventive dental medications, antibiotics and/or steroids for 1 week before the study were excluded. Written informed consents for participation in the clinical study of its result were obtained for all participants before the beginning of the study.

### Tested toothbrushes

**Fig. 1:**
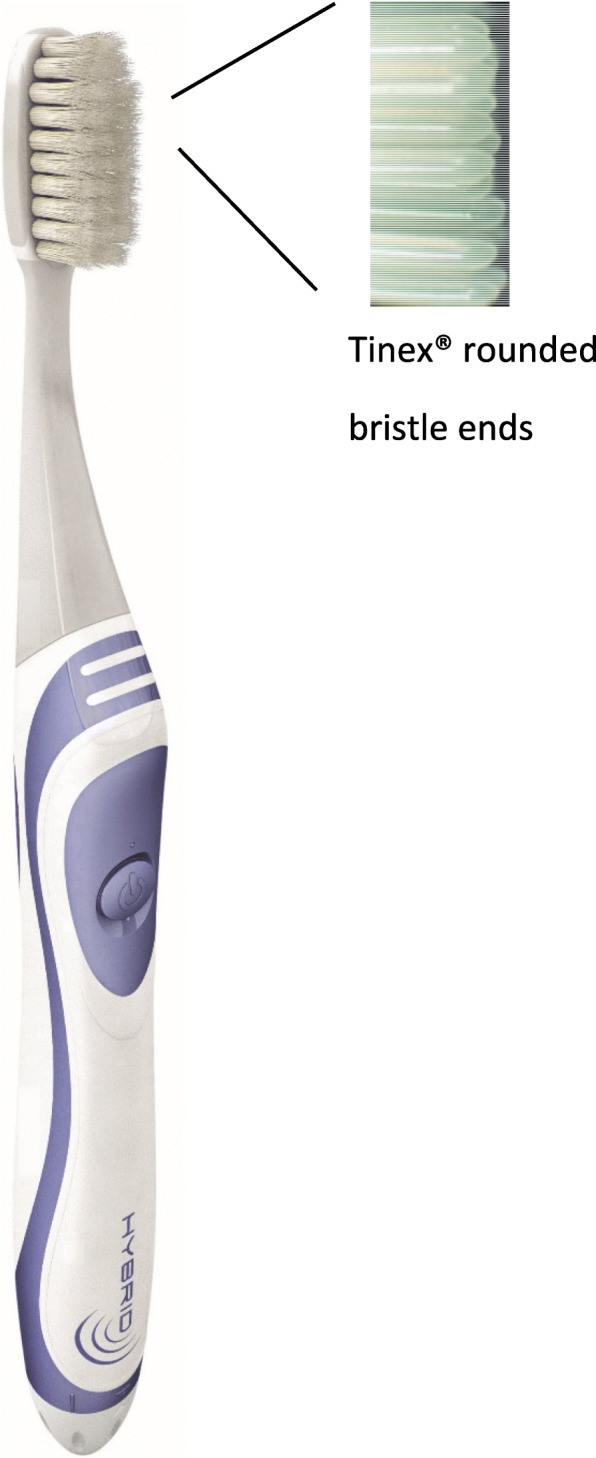
hybrid toothbrush (*Elgydium Clinic/Inava Hybrid*– Pierre Fabre Oral Care)

> Hybrid toothbrush (*Elgydium Clinic/Inava Hybrid*– Pierre Fabre Oral Care): this toothbrush looks like a manual toothbrush with a traditional oval brush head shape associated to sonic technology (Fig. 1). It weights around 1.59 oz. The toothbrush neck is thin and flexible. The bristles have a conical design (18/100 at the basis and 1/100 at the tip) which increase softness. They are made of Tinex® fibers with rounded ends, offering high flexibility for a non-traumatic brushing of gums and enamel. The brushing technology can be chosen among three modes: manual, sonic or combined (manual and sonic). The sonic mode uses vibration technology; the brush head makes side-by-side movements and produces up to 28,000 strokes per minute for an effective plaque removal.

The instructions for use given to the subjects were the following (combined mode):

1. *Wet the toothbrush (brush bristles) and apply a small amount of toothpaste.*
2. *Place the brush bristles in contact with the tooth with a 45 ° inclination to the gums.*
3. *Turn the toothbrush on once in the mouth to activate the sonic mode.*
4. *Apply slight pressure by slowly moving the brush head with a light circular motion.*
5. *Keep an inclination (45 °) and constant contact with the teeth during brushing.*
6. *Be sure to clean all surfaces of your teeth; do not forget your tongue.*
7. *Brushing time: two minutes.*

**Fig. 2:**
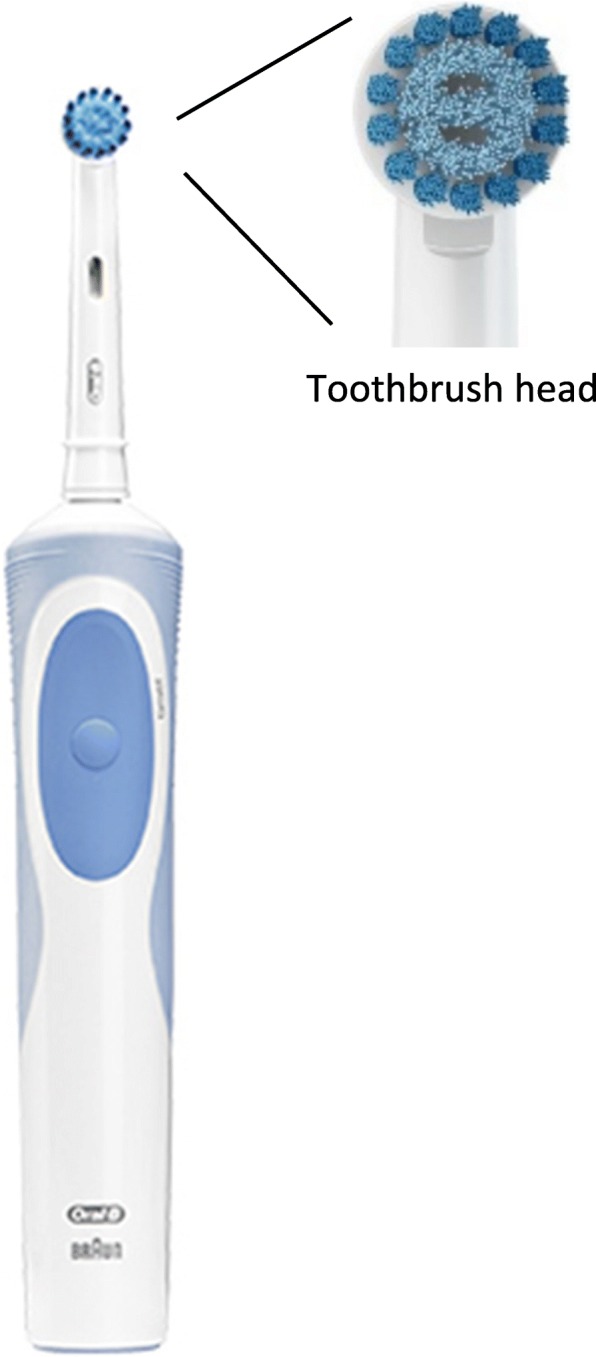
oscillating-rotating powered toothbrush (Oral-B Vitality 2D Sensitive Clean - Procter & Gamble)

> Oscillating-rotating toothbrush (Oral-B Vitality 2D Sensitive Clean - Procter & Gamble): this rechargeable electric toothbrush possesses a small circular brush head, with soft and highly flexible bristles convenient for sensitive gums (Fig. 2). It weights approximately 5.29 oz. This toothbrush uses rotation-oscillation action (=rotary technology); the brush head spins in a motion and makes 16 degree movements. It performs 7600 oscillations per minute. The instructions for use given to the subjects were the following:

1. *Wet brush head and apply a small amount of toothpaste. To avoid splashing, guide the brush head to your teeth before switching on the appliance.*
2. *Guide the brush head slowly from tooth to tooth, spending a few seconds on each tooth surface. Brush the gums as well as the teeth, first the outside, then the inside, finally the chewing surfaces. Do not press too hard or scrub. Do not forget to brush your tongue.*
3. *Brushing time: two minutes.*

### Trial schedule

On the day of the study, the subjects came to the laboratory after refraining from all oral hygiene procedures for 24 h and without eating, drinking and smoking for the last 4 hours before the visit. The subjects read, signed and dated the information sheet (instructions on the product use and restrictions related to the study) and informed consent forms in duplicate. After a verification of the inclusion and non-inclusion criteria, the dentist performed a clinical examination of the state of the oral cavity. Only the subjects with a” Silness and Löe Plaque Index” (PI) score between 1.0 and 2.0 and “Modified Gingival Index “(MGI) score between 1.0 and 2.0 were included in the study. After randomization, each participant received instructions on how to use the assigned toothbrush device. The same toothpaste was provided to all the subjects. A single brushing with the studied product (hybrid toothbrush) or the comparative product (oscillating-rotating device) was performed for exactly 2 minutes under supervision to ensure compliance with the manufacturer’s usage instructions. Immediately after brushing, another clinical examination of the state of the oral cavity was performed by the dentist and another PI scoring was done. Possible adverse reactions were noted. For this post-brushing evaluation, the dentist was blinded as to the device used. The participants completed a self-assessment questionnaire of the products’ acceptability after the first use.

### Evaluation tools

> “Silness and Löe Plaque Index” (PI) [25]: this assessment is based on recording, on all natural teeth, the thickness of the plaque at the gingival margin rather than its coronal extent on the tooth surface area. The scoring system goes from 0 to 3 (**0**_no plaque / **1**_deposit of invisible plaque but can be removed by a curette / **2**_deposit of plaque covering ^1^/_3_ of the cervical / **3**_deposit of plaque in abundance (more than ^1^/_3_ of the cervical)]. The index for the subject was obtained by summing the indexes for all surfaces (lingual and labial) of natural teeth and dividing by the number of surfaces examined. Variations (before brushing - after brushing) were calculated and statistics were performed to determine the significance of any variation. The device was considered effective if it induced a significant decrease in the score of the plaque.

> “Modified Gingival Index” (MGI) devised by Lobene et al. [26]: gum status was evaluated using a score ranging between 0 to 4 (**0**_absence of inflammation/ **1**_mild inflammation or with slight changes in color and texture but not in all portions of gingival marginal or papillary / **2**_mild inflammation, such as the preceding criteria, in all portions of gingival marginal or papillary / **3**_moderate, bright surface inflammation, erythema, oedema ^and^/_or_ hypertrophy of gingival marginal or papillary / **4**_severe inflammation: erythema, oedema ^and^/_or_ marginal gingival hypertrophy of the unit or spontaneous bleeding, papillary, congestion or ulceration). The MGI was scored for selected teeth (12, 16, 24, 36, 32 and 44). The scores of the four areas (buccal/lingual/mesial/distal) of the tooth were summed and divided by four to give the MGI for the tooth. For each subject, the MGI was obtained by adding the values of each tooth and dividing by the number of teeth assessed.

> Tolerance assessment: before and after the first brushing, an examination of the subject’s oral cavity was performed by the dentist to assess, either on soft tissues (gums, tongue, lips, palate) or hard tissues (teeth), the following signs:

- clinical signs: ulceration, desquamation, dyschromia, erythema, bleeding, papules, edema, cheilitis or other on soft tissues; dyschromia, tooth decay or other on hard tissues.
- functional signs: pruritus, stinging, pain, burning sensation, change in the quantity of saliva, oral dysesthesia, taste perversion, sensation of discomfort or other on soft tissues; dyschromia or other on hard tissues.

For each sign, the intensity was scored as: **0**_none / **1**_very mild / **2**_mild/ **3**_moderate.

A global tolerance assessment of each powered toothbrush was done on all the subjects who used the device at least once. The number and percentage of reactions related to each toothbrush was collected and the final tolerance assessment was estimated either as: Excellent (no functional nor physical signs related to the study) product observed or reported by the subjects) / Very good / Good / Moderate / Bad.

### Statistics

The raw variations (Δ) of the different studied parameters were calculated according to the following formulas:

$$ \Delta =\left({\mathrm{PI}}_{\mathrm{t}1}-{\mathrm{PI}}_{\mathrm{t}0}\right). $$

with: PI: Silness and Löe Plaque Index; t0: before brushing; t1: after brushing.

The descriptive statistics for quantitative data were computed for each time point, for each surface (lingual and labial) as well as for the change between (t1-t0).

To assess the effectiveness of the device after brushing, a paired t-test was applied on the change outcome (t1-t0). The normality assumption was checked with a Shapiro-Wilk test (α = 0.01). The type I error probability (α) was set at 5% in bilateral mode. The software used was Microsoft Excel® 2010 and SAS® v9.2.

## Results

A total of 66 subjects participated in the study (33 per group). The baseline demographics of the randomized subjects are given in Table 1.

**Table 1:** Baseline demographics of randomized subjects

		Hybrid (*n* = 33 subjects)	Oscillating-rotating (*n* = 33 subjects)
Age (years)	Mean (±SEM)	27.5 (±5.6)	30.2 (±7.4)
	Range	18–42	19–55
Gender	Male	14	6
	Female	19	27
LSPI	Mean (±SEM)	1.2 (±0.0)	1.2 (±0.0)
MGI	Mean (±SEM)	1.3 (±0.0)	1.2 (±0.0)

### Evaluation of plaque removal efficacy

Mean values of PI scores before and after a single brushing with either powered toothbrush are presented in Table 2.

**Table 2:** evolution of mean PI after a single brushing for the hybrid and oscillating-rotating powered toothbrushes; comparison of devices’ efficacy

PI	Device	Variation ∆ (t1-t0) (mean ± SEM)	∆%	Significance (paired t-test)	% of subjects with a positive effect
Global score	Hybrid	−0.5 ± 0.0	−45%	*p* < 0.0001	100%
	Oscillating-rotating	−0.5 ± 0.0	−43%	*p* < 0.0001	100%
	*Comparison*	*0.0 ± 0.2*		*p = 0.5474*	*(unpaired t-test)*
Labial side	Hybrid	−0.6 ± 0.0	−53%	*p* < 0.0001	100%
	Oscillating-rotating	−0.6 ± 0.0	− 52%	*p* < 0.0001	100%
	*Comparison*	*0.0 ± 0.2*		*p = 0.9273*	*(unpaired t-test)*
Lingual side	Hybrid	−0.5 ± 0.0	−37%	*p* < 0.0001	100%
	Oscillating-rotating	−0.4 ± 0.0	−34%	*p* < 0.0001	100%
	*Comparison*	*−0.05 ± 0.2*		*p = 0.3022*	*(unpaired t-test)*

Under these study conditions, after a single use, the hybrid toothbrush presented a global anti-plaque efficacy characterized by a significant decrease of the global PI of 45% on average (*p* < 0,0001; paired t-test); this effect was observed in 100% of subjects. The oscillating-rotating toothbrush used under the same conditions showed a similar efficacy: reduction of global PI of 43% on average (*p* < 0.0001; paired t-test); effect observed in 100% of subjects.

For each tooth brushing technique, removal of plaque was more effective on the labial side than on the lingual side (mean reduction of PI of 53% versus 37% respectively for the hybrid toothbrush and 52% versus 34% for the oscillating-rotating device). However, whatever the side and the device, the decrease of plaque index was statistically significant and observed on the totality of the participants.

The comparison of methods showed that the hybrid toothbrush (manual and sonic combination) was as effective as the oscillating rotating one in plaque removal after a single use (*p* > 0.05; unpaired t-test).

### Global tolerance assessment

> With the hybrid toothbrush, 15 subjects (45%) presented reactions with causality assessment “likely or very likely.” The most common reaction was bleeding (12 subjects, with very mild or mild intensity).

> With the oscillating-rotating powered toothbrush, 14 (42%) subjects presented reactions with causality assessment “likely or very likely.” For 13 out of 14 participants, the reaction was bleeding with very mild or mild intensity.

However, no subject was withdrawn of the study due to theses reactions. Subjects neither modified methods of brushing nor temporally stopped the brushing due to the reactions.

According to the investigator, bleeding reactions could be due not only to the toothbrushes (bristles), but also to the fact that the participants used this type of toothbrush (powered) for the very first time. As the teeth brushing was done under supervision, the subjects could have brushed too intensively… Considering this, the dentist judged the global tolerance of both powered toothbrushes as “Good.”

### Global appreciation of the powered toothbrushes

**Fig. 3:**
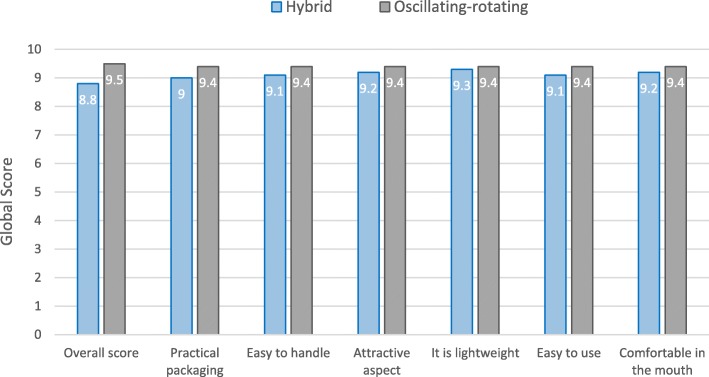
mean global appreciation of the hybrid and the oscillating-rotating powered toothbrushes. Overall score was scored on a scale ranging from 0 (really dislike) to 10 (really like). Affirmations were scored on a scale ranging from 0 (totally disagree) to 10 (totally agree)

**Fig. 4:**
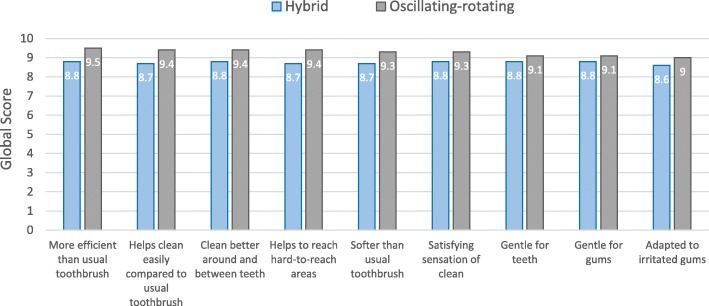
mean global appreciation of the hybrid and the oscillating-rotating powered toothbrushes. Affirmations were scored on a scale ranging from 0 (totally disagree) to 10 (totally agree)

The participants completed a subjective evaluation questionnaire after their first use. A summary of the answers is presented in Figs. 3 and 4.

> The hybrid toothbrush was appreciated by the majority of the subjects for its characteristics; its intensity of vibration was just about right for 91% of the participants. The new hybrid sonic toothbrush was judged better than the usual manual one for 87% of the subjects.

> The oscillating-rotating toothbrush was also liked by the majority of the subjects; its intensity of vibration was considered just about right for 94% of the participants. 90% of the subjects rated the marketed oscillating-rotating toothbrush better than their usual manual one.

## Discussion

The maintenance of periodontal health requires supragingival dental plaque removal. Tooth brushing is a key element in mechanical plaque control [6]. Several randomized, controlled clinical trials recognized a superiority of powered over manual toothbrushes in removing dental plaque [19, 20]. Without distinction on the type of powered toothbrushes used, an overall benefit of 11% reduction in plaque was shown at one to 3 months and 21% at longer than 3 months [19]. With regards to gingivitis, a 6% reduction was shown at one to 3 months and an 11% over the long term. In 2010, different power toothbrush technologies were compared for plaque and gingivitis control [27]. The authors concluded that, over a period of four to 12 weeks, brushes with a rotating-oscillating action appeared more effective than sonic ones for plaque and gingivitis reduction. However, they noticed that the difference was small and its clinical importance, unclear. A recent review on the efficacy of powered toothbrushes following a single-use test highlighted the contribution of several factors to the observed efficacy on dental plaque: the power supply (rechargeable or replaceable battery), the mode of action, the brushing duration as well as the type of instructions [28]. The magnitude of the outcome was also highly dependent on the index scale used to score plaque for the evaluation.

These conflicting results may have contributed to the resistance of a portion of the population to becoming powered toothbrush users. Some consumers stay attached to their manual brushing experience, favor a traditional brush head characteristic and the ability to brush several teeth at once. Modifying oral hygiene habits is difficult to achieve and maintain over time [29]. Moreover, some of the powered toothbrush users sometimes return to their manual brush, either because of a vacation period, dead battery or hybrid usage (for example, manual tooth brushing in the morning and powered one in the evening) [30]. Based on these observations, the concept of a hybrid (manual and sonic) toothbrush emerged: the new brush evaluated in the present study was designed as a manual toothbrush with the addition of sonic technology. The final product is light (it weights three times less than the comparative marketed powered oscillating-rotating toothbrush), which can be an advantage for children or the elderly. It is space-saving since it does not need any electrical base. It is easy to carry and has a battery life of 1 month. The brush head needs to be replaced every 3 months, like any other toothbrush. “Hybrid” toothbrush means that it can be used either in manual mode, in sonic mode or in a combined mode (manual and sonic). Since the brush bristles are made of highly flexible Tinex® fibers, conically designed and rounded ended, a non-traumatic brushing of gums and enamel can be performed. Such bristles suit particularly well to sulcular tooth brushing. In this technique, the brush is positioned at about 45 degrees to the tooth and the tooth brush bristles are pushed into the sulcus [31]. The brush is then moved back and forth and removed by a downward and outward movement in the top arch. The toothbrush is moved to another tooth and the process is repeated. People presenting gum sensitivity can benefit from this gentle brush. In the manual mode, the user can keep his familiar brushing technique; in the sonic mode, the “non-contact” brushing linked to fluid dynamics completes the soft mechanical scrubbing. Sonic toothbrushes used in combination with a fluoride toothpaste demonstrate significantly less interproximal plaque and deliver significantly higher concentration of fluoride in that plaque, compared to manual or oscillating-rotating toothbrushes [32]. A standardized in vitro test -performed on a phantom tooth model cleaned by a robot proved that the hybrid toothbrush (combined mode) eliminates ten times more plaque in the approximal spaces compared to a conventional ADA manual toothbrush (internal data). Brushing exercise such as the one reported in the present study, proved that the plaque removal ability of the hybrid toothbrush used in the combined mode is as good as an oscillating-rotating one, considered as the gold standard mode. The percentages of plaque reduction obtained (around 45%) are very similar to the one estimated by Rosema et al. for powered toothbrushes following a brushing exercise (46% on average) [28]. The tolerance of the new toothbrush used in the combined mode was good as well as its overall appreciation. In order to investigate the improvement of gingival health, longer term studies should be performed in the future.

## Conclusion

The results of this one-time use trial demonstrate that the hybrid toothbrush (used in the combined mode) is as good as a marketed oscillating-rotating toothbrush for plaque removal. The hybrid technology -offering the choice between either the traditional manual brushing technique, the sonic mode or the combined mode- allows each user to adapt tooth brushing to his desire, his skills or his mouth condition. We hypothesize that such an individualized approach can favor long term compliance with oral health recommendations and improve global oral wellness [33].

## Abbreviations

ADA: American Dental Association
MGI: Modified Gingival Index
PI: Silness and Löe Plaque Index
